# An integrated network analysis, RNA-seq and *in vivo* validation approaches to explore the protective mechanism of Mongolian medicine formulae Ruda-6 against indomethacin-induced gastric ulcer in rats

**DOI:** 10.3389/fphar.2023.1181133

**Published:** 2023-08-10

**Authors:** Lan Feng, Lisha A., Terigele Bao, Xiyele Mu, Na Ta, Qiang Duan, La Ta, Yongsheng Chen, Laxinamujila Bai, Minghai Fu

**Affiliations:** ^1^ Key Laboratory of Tropical Translational Medicine of Ministry of Education, Hainan Provincial Key Laboratory for Research and Development of Tropical Herbs, School of Pharmacy, Hainan Medical University, Haikou, China; ^2^ NMPA Key Laboratory for Quality Control of Traditional Chinese Medicine (Mongolian Medicine), School of Mongolian Medicine, Inner Mongolia Minzu University, Tongliao, China; ^3^ Key Laboratory of Castor Breeding of the State Ethnic Affairs Commission, Inner Mongolia Minzu University, Tongliao, China; ^4^ The Second Clinical Medical College of Nanchang University, Nanchang, China

**Keywords:** traditional Mongolian medicine, Ruda-6, gastric ulcer, indomethacin, network analysis, RNA-seq

## Abstract

Gastric ulcer (GU) is one of the most prevalent digestive diseases that seriously affects people’s health. Previous studies have demonstrated the anti-GU effect of Ruda-6 (RD-6), a classic formulae of traditional Mongolian medicine. However, the underlying mechanism of RD-6 against GU remains elusive. Thus, we conducted an integrative approach of network analysis, RNA-seq, and *in vivo* validation experiment to elucidate the therapeutic mechanisms of RD-6 in preventing GU. A network analysis was performed to predict the potential targets of RD-6. Rats were pretreated with RD-6 at different doses for 21 days, followed by GU induction with indomethacin injection. The ulcer index and inhibition rates were calculated, and the levels of inflammatory related factors were determined by ELISA. The gastroprotective mechanism of RD-6 against ulceration was verified by RNA-seq and the key pathway was detected by *in vivo* validation. As the network analysis predicted, RD-6 exerts anti-GU effects by regulating 75 targets and 160 signaling pathways. Animal experiment results suggested that pretreatment with RD-6 significantly ameliorated the gastric mucosal injury and inflammatory response, as evidenced by a reduced ulcer index, decreased interleukin (IL)-1β, IL-6, and IL-17 levels, and increased prostaglandin E2 (PGE2) levels in the GU model rats induced by indomethacin. RNA-seq data identified four potential hub genes that were primarily involved in the IL-17 signaling pathway. Furthermore, *in vivo* validation experiment showed that RD-6 inhibited the IL-17 signaling pathway by down-regulating the expression of IL17RA, proto-oncogene C-Fos (FOS), IL1B and prostaglandin-endoperoxide synthase 2 (PTGS2). Taken together, the present study provides evidence that RD-6 could effectively protect against indomethacin-induced GU, which might be attributed to suppressed inflammation. The IL-17 signaling pathway may be one of the crucial mechanisms that mediates the effect of RD-6.

## Graphical Abstract

**Figure:**
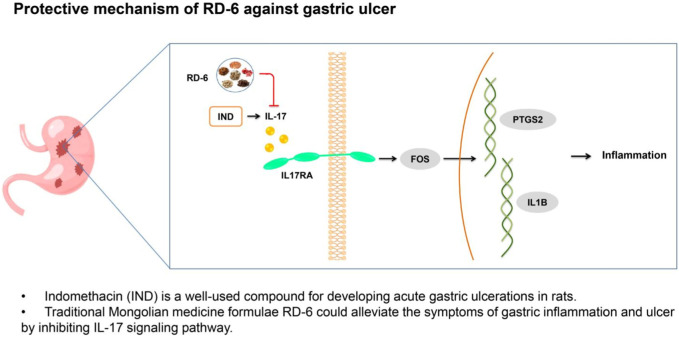

## 1 Introduction

Gastric ulcer (GU), a common and frequent gastrointestinal disease occurring on the gastric mucosa, seriously affects about 10%–15% of the population in the world (Liu et al., 2022). The occurrence of GU is mainly driven by aggressive factors such as *Helicobacter pylori* infection, non-steroidal anti-inflammatory drug (NSAID) overdoses, excessive alcohol consumption, stress, etc. (Laine et al., 2008; Yegen, 2018). Gastric mucosal injury is closely related to an inflammatory reaction. These destructive factors directly contact the gastric mucosa, causing inflammatory cell infiltration and activating the transcription of various pro-inflammatory mediators, thus triggering the gastric lesions (Datta De and Roychoudhury, 2015; de Souza et al., 2019; Liu et al., 2022). Currently, various drugs, including ranitidine, omeprazole and sucralfate are available for treating GU in clinical applications. However, considering the problems such as adverse side effects, drug interactions, bacterial resistance caused by these drugs (Khayyal et al., 2019; Zhang et al., 2022), researchers are increasingly interested in traditional medicine (Bao et al., 2022; Liu et al., 2022). Botanical drugs have been used for thousands of years. Because of their multi-component nature and low toxicity, they provide an alternative method for the treatment of GU.

**FIGURE 1:**
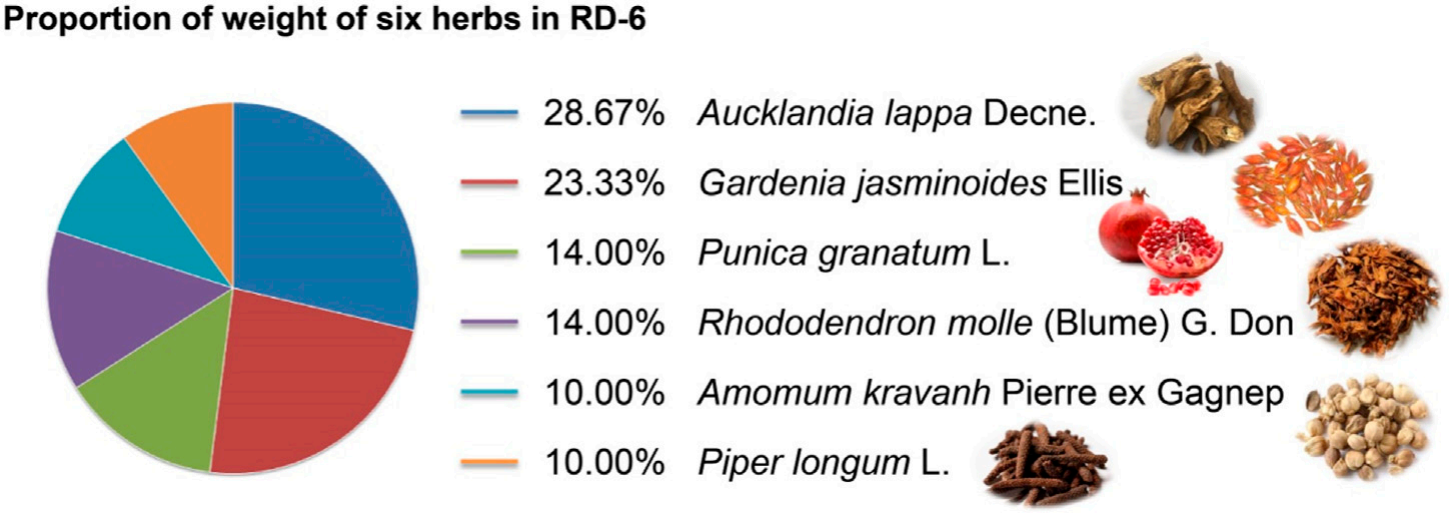
The weight proportion of RD-6 formulae.

Mongolian medicine Ruda-6, also known as Liuwei Muxiang Powder in the China Pharmacopoeia (ChP, 2020), is a traditional formulae consisting of Ruda (roots of *Aucklandia lappa* Decne.), Zhurura (fruits of *Gardenia jasminoides* Ellis), Anar (whole pomegranate of *Punica granatum* L.), Hvrch-chagan-checheg [flower of *Rhododendron molle* (Blume) G. Don], Sugmel (fruits of *Amomum kravanh* Pierre ex Gagnep), and Bibiling (near-mature ears of *Piper longum* L.) (Figure 1). RD-6 was documented in the “Four Tantras of Nectar” (about the 18th century) (Ixi, 2017) (p. 46), a Mongolian medicine masterpiece, as a well-known prescription for treating gastrointestinal disorders. Clinical trials have shown that RD-6 exhibits an available gastro-protective effect on acute and chronic gastritis, spasms, gastro-duodenal ulcers, etc., due to its traditional effects of opening depression, promoting Qi and relieving pain (Dao and Wang, 2017; Ge, 2017). In addition, RD-6 has been found to inhibit the stomach mucosal damage of different types of GU animal models, including stress-induced, acetic acid-induced, reserpine-induced, ethanol-induced, pyloric ligation-induced, etc. (Bai et al., 1998). Our previous study has shown that RD-6 has a beneficial effect on the imbalance of metabolites and intestinal microorganisms in rats caused by indomethacin (Feng et al., 2023). On the other hand, Ruda (the primal medicine of RD-6) has been proven to have an anti-GU effect (Xu et al., 2020), and the other adjuvant medicines also have potential for gastric protection (Ju, 2009; Lai et al., 2009). However, the molecular mechanism of the anti-GU effect of RD-6 has not been fully elucidated, and further research is needed.

As a novel approach to analyzing drug mechanisms based on the concept of “drug-target-disease,” network analysis could identify multiple components, multiple targets and multiple pathways, which provide a strategy to understand the intervention mechanisms of drugs and disease (Wang et al., 2019; Zhang et al., 2019). On the other side, RNA-sequencing (RNA-Seq) technology is widely used to acquire the transcriptomic profiles of different physiological and pathological conditions, and to study the molecular mechanisms of drug action (Liu et al., 2021; Bao et al., 2022). In this paper, we conducted an integrative network analysis and RNA-seq study to clarify the therapeutic mechanisms of RD-6 in preventing GU. Firstly, the potential active components, key targets and mechanisms of RD-6 against GU were predicted through network analysis. Then, the anti-inflammatory effect of RD-6 was confirmed *in-vivo* using the indomethacin-induced GU rat model. Furthermore, the mechanism of RD-6 anti-GU was explored by RNA-Seq, and the related targets were confirmed by quantitative real-time PCR (qRT-PCR) and western blotting (WB) detection. Taken together, this study provides evidence that RD-6 can prevent indomethacin-induced GU by alleviating inflammation.

## 2 Materials and methods

### 2.1 Chemicals and reagents

Commercial Ruda-6 (Inner Mongolia MengWang Pharmaceutical Co., Ltd., Inner Mongolia, China; Lot# 2104053) and ranitidine (Foshan Chiral Pharmaceutical Co., Ltd., Guangdong, China; Lot# 2101532) were purchased from a pharmacy. Indomethacin (Lot# 088M4033V) was obtained from Sigma-Aldrich (St. Louis, MO, United States). The kits used to determine rat IL-1β (Cat.# JM-01454R1), IL-6 (Cat.# JM-01597R1), and PGE2 (Cat.# JM-01475R1) were from Jingmei Biotechnology Co., Ltd. (Jiangsu, China). Rat IL-17 detection kit (Cat.#F2964) was provided by FANKEW Biotechnology Co., Ltd. Total RNA extraction kit (Lot# X0718) was from Tiangen Biotech Co., Ltd. (Beijing, China). The RNA-seq library construction kit (code No.: NR605) was provided by Vazyme Biotech Co., Ltd. (Nanjing, China). The RNA sequencing kit (Lot# 20425496) was obtained from Illumina (San Diego, CA, United States). The first strand complementary DNA was synthesized with a PrimeScript™ 1st strand cDNA synthesis kit (TaKaRa, Japan; Lot# AM60715A). qRT-PCR reactions were performed using TB Green^®^ Premix Ex TaqTM (Tli RNaseH Plus) (TaKaRa, Japan; Lot# ALG 1981A). Rabbit antibody against IL17RA (Cat.# PAB30301) was purchased from Bio-swamp Life Science Lab (Wuhan, China). Horseradish peroxidase (HRP) conjugated goat anti-rabbit secondary antibody (Cat.# BA1054) and rabbit antibodies against FOS (Cat.# BA0207-2), PTGS2 (Cat.# A00084), IL1B (Cat.# PB0055) and β-actin (Cat.# BA2305) were bought from BOSTER Biological Technology Co., Ltd. (Wuhan, China).

### 2.2 Network analysis

#### 2.2.1 Active compounds and target prediction

RD-6 powder was extracted with ultrapure water and utilized to identify the components through High-Performance Liquid Chromatography with Tandem Mass Spectrometry (HPLC-MS/MS). In all, 284 chemical ingredients were acquired (Supplementary Table S1). The potential active compounds of RD-6 were then selected with oral bioavailability (OB ≥ 30%) and drug-likeness (DL ≥ 0.18) as criteria based on the Traditional Chinese Medicine System Pharmacology Database and Analysis Platform (TCMSP, http://tcmspw.com/tcmsp.php) (Ru et al., 2014). In addition, costunolide and dehydrocostus lactone were also included in this study as they are the effective components of the main drug of RD-6, Muxiang (Aucklandiae Radix) (ChPC, 2020; Huang et al., 2021). The corresponding targets of these compounds were collected from the TCMSP platform. Then the obtained targets were converted into gene names through the Uniprot (http://www.uniprot.org/) database, and the unmatched genes were eliminated. The detailed components and targets of RD-6 are shown in Supplementary Tables S2–S3.

Meanwhile, GU related genes were screened from GeneCards (https://www.genecards.org/) (Stelzer et al., 2016), Online Mendelian Inheritance in Man (OMIM, https://omim.org/) (Amberger et al., 2015), PharmGkb (https://www.pharmgkb.org/) (Gong et al., 2008), TTD (http://db.idrblab.net/ttd/) (Zhou et al., 2022) and DrugBank (https://go.drugbank.com/) (Wishart et al., 2018) databases (Supplementary Table S4). The overlapping targets were further checked and duplicate targets were removed.

#### 2.2.2 The protein-protein interaction (PPI) network construction

The intersection of the targets of RD-6 and GU was taken to obtain the potential therapeutic targets of RD-6 against GU. The corresponding targets were uploaded to the STRING (https://cn.string-db.org/) (Szklarczyk et al., 2019) database for PPI network analysis, with “*Homo sapiens*” genes as a requisite and a confidence score ≥0.7. The TSV-format file was downloaded and then hub genes were screened with the assistance of the Cytoscape3.8.0 software. The number of connections between each node in the network was expressed by the degree value.

#### 2.2.3 KEGG pathway enrichment

The corresponding targets were then imported into the Metascape database (https://metascape.org/gp/index.html#/main/step1) (Zhou et al., 2019) to perform Kyoto Encyclopedia of Genes and Genomes (KEGG) pathway enrichment analysis. The organism was set as *H. sapiens*. A threshold of *p*-value was set to below 0.05. The results of KEGG pathway enrichment analysis were arranged according to their significance and presented as a bubble plot.

#### 2.2.4 The “component-target-disease” network construction

The active compounds in RD-6 and the potential targets against GU were imported into the Cytoscape 3.8.0 software to construct a “compound-target-disease” network and draw the network analysis figure of RD-6.

### 2.3 Animal experiments

#### 2.3.1 Animals

Male Sprague-Dawley (SD) rats (weighting 200.0 ± 20.0 g) were obtained from Changsheng Biotechnology Company (Changchun, China). Animal experimental procedures were approved by the Animal Ethical Committee of Inner Mongolia Minzu University (Ethics number: NM-LL-2021-06-15-1). All rats were housed in a controlled environment (temperature 24°C ± 2°C, humidity 50% ± 5% and a 12 h dark-light cycle) with free access to food and water. Animals were adaptively reared for 7 days before the experiments.

#### 2.3.2 Drug administration and sample collection

Rats were randomized into six groups (*n* = 10) including the normal control (NC) group (pretreated with deionized water), the indomethacin-induced model (IND) group (pretreated with deionized water), the ranitidine (RAN) group (40 mg/kg), as well as the RD-6-pretreated group at three concentrations (RD-6-L/M/H). As suggested in the China Pharmacopoeia (ChP, 2020), the dosage of Ruda-6 is 42.857 mg/kg for humans, which is equal to 0.27 g/kg for rats. Therefore, a low (0.27 g/kg), middle (1.35 g/kg) or high (2.7 g/kg) dose of RD-6 was given to the rats. All drugs were dissolved and suspended in deionized water and orally administered daily for three consecutive weeks. Rats were fasting for 24 h before the indomethacin challenge. On the 21st day, gastric ulceration was induced by oral administration of 30 mg/kg indomethacin (suspended in 0.5% CMC-Na) for all groups except the control group (Ogaly et al., 2021). After 6 h, all rats were anesthetized with a 50 mg/kg pentobarbital sodium injection. For further analysis, the stomach was excised, cut along the great curvature, gently cleaned in cold saline, expanded and photographed, and stored at −80°C.

#### 2.3.3 Evaluation of the gastric ulcer

The evaluation of ulcer index was performed as previously described scoring method: briefly, no lesion (score 0), hemorrhagic spot (score 1), lesion ≤1 mm (score 2), 1 mm < lesion ≤2 mm (score 3), 2 mm < lesion ≤4 mm (score 4), 4 mm < lesion (score 5), and twice for width >1 mm (Guth et al., 1979). Besides, further objective characterization of GU was carried out with ImageJ software to measure and evaluate the ulcer area (mm^2^). The ulcer inhibition rate was calculated as follows: ulcer inhibition rate = (ulcer area (model) - ulcer area (treated))/ulcer area (model) × 100%. After evaluating the ulcer index, gastric tissue was divided into two parts: left and right. In subsequent techniques, gastric tissue at the same location was randomly selected from each experimental group.

#### 2.3.4 Histological examination

The rat stomach samples were immersed in 4% paraformaldehyde, followed by paraffin embedding, cut into 5 μm sections, and stained with hematoxylin and eosin (H&E). Pathological changes in stomach tissues were evaluated by light microscope (Nikon Eclipse E100).

#### 2.3.5 ELISA analysis

The gastric tissue was cut, weighed and homogenized in a corresponding volume of cold PBS (1 g: 9 mL). Then the homogenate was centrifuged at 5,000 r/min for 10 min at 4°C to collect the supernatant. The levels of IL-1β, IL-6, IL-17, and PGE2 in gastric tissue were determined with an ELISA kit following the manufacturer’s instructions. The optical densities were measured at 450 nm. Graphpad Prism 8.0 software was used for graphics.

### 2.4 RNA-seq analysis

Total RNA from gastric tissue was isolated and purified using the Total RNA Extraction Kit (Tiangen, China). The concentration and purity of the total RNA were quantified using infinite M200 pro (Tecan), and integrity was assessed by 2% agarose gels. A total of 1 μg RNA was used as input material for the RNA sample preparations. Sequencing libraries were generated using the VAHTS^®^ Universal V8 RNA-seq Library Prep Kit for Illumina (Vazyme, China), following the manufacturer’s instructions, and index codes were added to attribute sequences. Libraries were then accurately quantified using the Equalbit^®^ 1 × dsDNA HS Assay Kit (Vazyme, China), and library integrity was assessed using the Agilent DNA 1000 Kit of the Bioanalyzer 2100 system (Agilent Technologies, United States). Qualified libraries were sequenced on the Illumina NextSeq 550 platform to obtain 150 bp paired-end data. The raw reads of fastq format were filtered by removing adapter and low-quality reads that contained poly-N sequences or inferior quality reads to obtain clean reads. The clean reads were then mapped to the *Rattus norvegicus* reference genome sequence using the HISAT2 tools. The expression levels of genes in each sample were estimated using the DESeq2 R Package (1.16.1), and the *p*-value and fold change were calculated. Genes with |Log2fold-change (FC)| ≥ 1 and an adjusted *p*-value <0.05 were assigned as differentially expressed genes (DEGs). The volcano plots and heatmap were generated with MetwareCloud online platform. The Metascape database [37] was used to perform the GO and KEGG enrichment analysis of the DEGs, and the pathways with a *p*-value below 0.05 were considered significantly enriched.

### 2.5 Quantitative real-time PCR validation

Rat-specific primers for candidate genes were purchased from Sangon Biotech Co., Ltd. (Shanghai, China) and primer sequences are presented in Supplementary Table S9. Approximately 1 μg of total RNA extracted from rat stomach tissue was reversely transcribed to first-strand cDNA by PrimeScript™ 1st strand the cDNA synthesis kit (TaKaRa, Japan). The real-time quantitative PCR was performed with the TB Green^®^ Premix Ex Taq™ (Tli RNaseH Plus) kit (TaKaRa, Japan) using the CFX96 Real-Time System (BIO-RAD, CA, United States). The reactions were designed in 20 μL volumes. The temperature program included a pre-denaturation step at 95°C for 3 min, followed by 40 amplification cycles: 95°C for 10 s, 60°C for 20 s, 72°C for 20 s. Each experiment was conducted three times. The 2^−ΔΔCT^ formula was used to calculate the relative expression levels, and β-actin was used as the internal reference. Results were expressed as a fold change relative to the normal control group.

### 2.6 Western blotting analysis

The rat gastric tissue was washed with cold PBS and homogenized using RIPA lysis buffer for 30 min. The concentration of total protein was measured using BCA protein assay kits. The processed protein samples (30 μg) were segregated by 10% sodium dodecyl sulfate polyacrylamide gel electrophoresis (SDS-PAGE) and transferred to a poly-vinylidene difluoride (PVDF) membrane after electrophoresis. Then the membranes were blocked with 5% nonfat milk to block the nonspecific interacting proteins for 1 h at room temperature. The following primary antibodies were applied to incubate the membranes overnight at 4°C: IL17RA (1:2,000), FOS (1:1,000), PTGS2 (1:1,000), IL1B (1:1,000), and β-actin (1:2,000). The membranes were swilled with TBST buffer and incubated with HRP-conjugated goat anti-rabbit secondary antibody (1:5,000) for 1 h at room temperature. Finally, the enhanced chemiluminescence (ECL) detection reagent was added, then the density of the respective protein bands was processed visually using the ChemiDoc™ instrument (BIO-RAD, CA, United States) and analyzed by ImageJ software. The expression levels of the proteins were normalized compared with β-actin.

### 2.7 Statistical analysis

The data are expressed as mean ± SEM. One-way ANOVA with the uncorrected Fisher’s LSD test was used to evaluate multiple comparisons. Values of *p* < 0.05 and *p* < 0.01 were considered to be statistically significant. GraphPad Prism 8.0 software was used to perform the analysis and graphing.

## 3 Results

### 3.1 Network pharmacological prediction of the anti-GU mechanism of RD-6

#### 3.1.1 Potential targets collection

**FIGURE 2:**
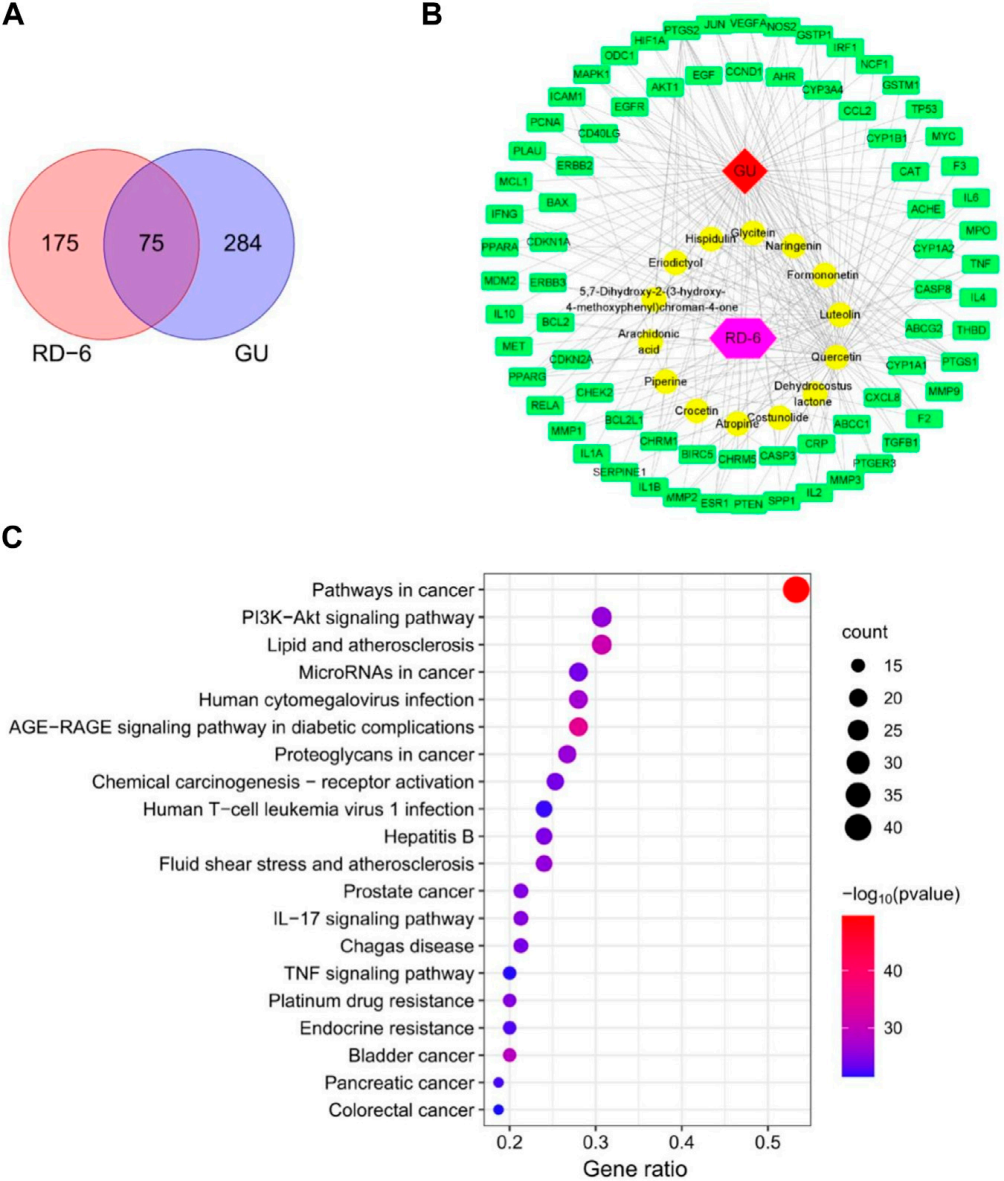
Network analysis prediction for the effect of RD-6 against GU. **(A)** Venn diagram of potential targets of RD-6 for anti-GU. **(B)** Compound-target network by Cytoscape 3.8.0. The red diamond represents the gastric ulcer; the yellow circle represents the potential active components of RD-6; the purple hexagon represents the Mongolian medicine formulae RD-6; the green rectangle represents the potential targets. **(C)** The top 20 significantly enriched KEGG pathways. The size of the circles represents the number of genes and the color represents the *p*-value.

A total of 12 active ingredients in RD-6 fulfilled the criteria of OB ≥ 30% and DL ≥ 0.18 according to our previous LC-MS/MS analysis. Besides, costunolide and dehydrocostus lactone did not meet the criteria, but they were also presented as candidates according to the literature. Hence, 14 main active compounds were considered as potential active constituents in RD-6 (Supplementary Table S2). These 14 active compounds were then used to identify the potential targets and finally acquired 250 targets of RD-6 from TCMSP (Supplementary Table S3). Meanwhile, 359 GU-related targets were screened from Gene-Cards, OMIM, PharmGkb, TTD and Drugbank databases after the removal of duplicates (Supplementary Table S4). Then the 250 compound targets and 359 disease targets were used to draw a Venn diagram. Finally, a total of 75 overlapping targets were obtained, which were considered as potential targets for RD-6 against GU (Figure 2A).

#### 3.1.2 PPI analysis

A PPI network was established to research the underlying mechanism of RD-6 against GU, and 10 targets were verified as potential hub targets based on the values of degree, including TNF (degree = 38), IL6 (degree = 37), AKT1 (degree = 35), JUN (degree = 35), TP53 (degree = 34), VEGFA (degree = 33), EGFR (degree = 30), IL1B (degree = 30), RELA (degree = 28), and CASP3 (degree = 28) (Supplementary Figure S1A). Furthermore, the 75 common targets were divided into 5 categories according to the MCODE function analysis, which were associated with regulation of cell death, cytokine activity, and inflammatory response, etc. (Table 1, Supplementary Figure S1B).

**TABLE 1:** Cluster analysis of the effect of RD-6 against GU based on MCODE.

MCODE	Score	Top 5 of GO	logP
MCODE1	11.63	response to UV	−22.48
		positive regulation of programmed cell death	−22.38
		positive regulation of cell death	−21.53
		positive regulation of apoptotic process	−20.71
		response to light stimulus	−20.28
MCODE2	9.67	cytokine activity	−13.83
		cytokine receptor binding	−13.34
		positive regulation of leukocyte cell-cell adhesion	−13.30
		inflammatory response	−12.92
		positive regulation of cell-cell adhesion	−12.73
MCODE3	5.60	long-chain fatty acid metabolic process	−14.71
		long-chain fatty acid biosynthetic process	−14.55
		xenobiotic metabolic process	−14.39
		estrogen 16-alpha-hydroxylase activity	−13.52
		cellular response to xenobiotic stimulus	−13.25
MCODE4	3.20	transmembrane receptor protein tyrosine kinase signaling pathway	−8.54
		gland development	−8.53
		positive regulation of chemotaxis	−8.14
		positive regulation of transferase activity	−8.05
		enzyme-linked receptor protein signaling pathway	−7.65
MCODE5	3.00	serine-type peptidase complex	−9.75
		positive regulation of blood coagulation	−9.15
		positive regulation of hemostasis	−9.15
		positive regulation of coagulation	−9.05
		positive regulation of wound healing	−8.08

#### 3.1.3 “Compound-target-disease” network and analysis

The 14 potential active compounds and 75 identified targets were used to construct a compound-target network (Figures 2B, Supplementary Figure S2). It can be seen that one compound in the RD-6 can be linked to more than one target. Among these active compounds, 6 active compounds, quercetin, luteolin, naringenin, formononetin, piperine and dehydrocostus lactone, had a higher number of connections. For example, quercetin was connected to 64 targets, and luteolin was connected to 33 targets. What’s more, some targets can be linked to more than one active compound. PTGS2 was targeted by 12 active compounds, and PTGS1 was targeted by 10 active compounds.

#### 3.1.4 Pathway analysis

To accurately analyze the possible anti-GU mechanism of RD-6, the KEGG pathway enrichment analysis was performed on 75 common targets. In total, 160 signaling pathways were identified (Supplementary Table S6), and the top 20 of those were selected for visual display (Figure 2C). According to the KEGG enrichment analysis, the targets were gathered in the PI3K/Akt, IL-17 and TNF signaling pathways.

### 3.2 RD-6 alleviated the gastric mucosal damage in indomethacin-induced GU rats

**FIGURE 3:**
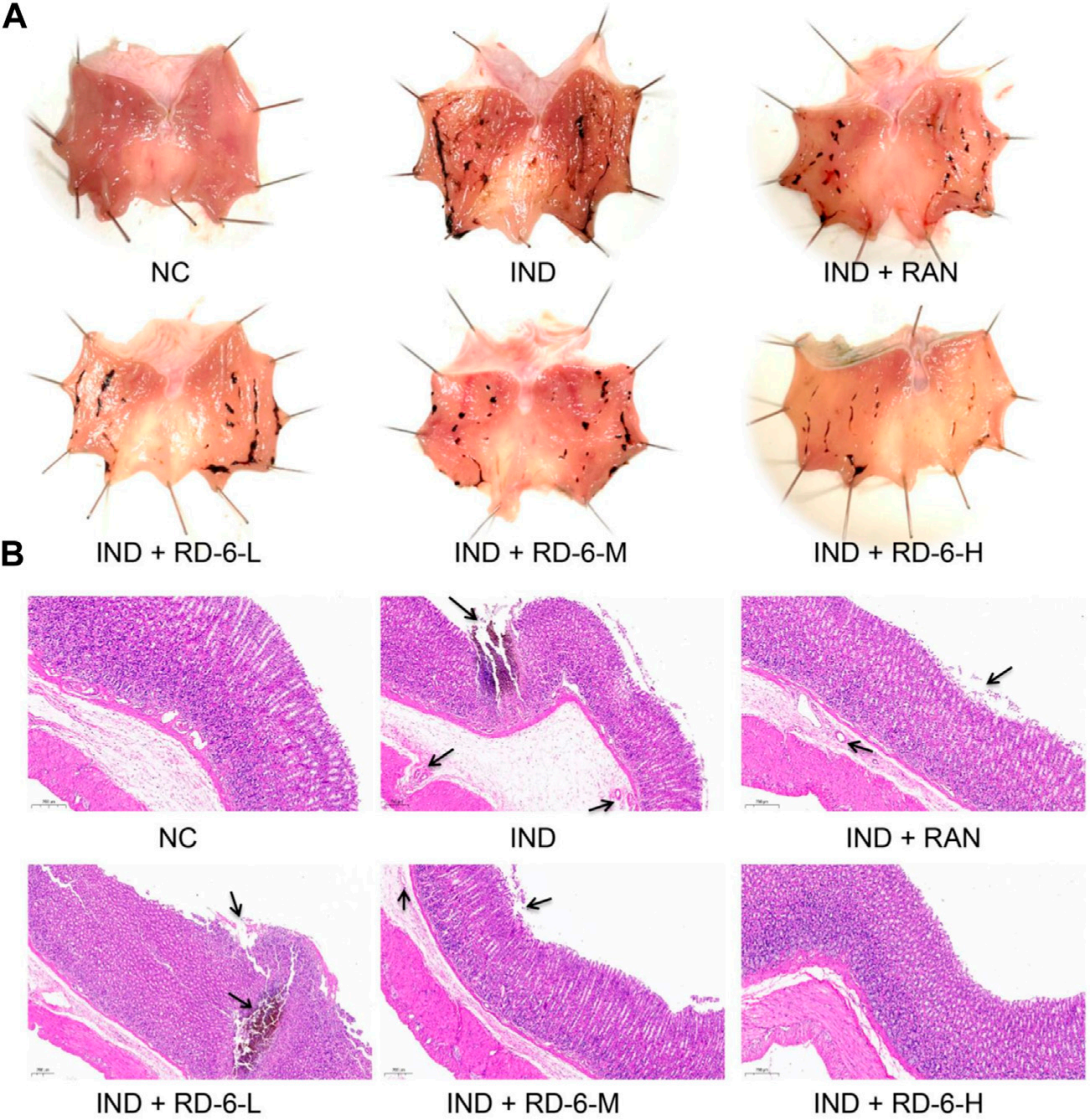
Histopathological features of gastric tissues after RD-6 pretreatment on IND-induced GU in rats. **(A)** Photographic images of rats in each group 6 hours after IND (30 mg/kg) induction. **(B)** Histological sections of gastric tissues stained with hematoxylin and eosin (H&E) in each group. NC, normal control; IND, indomethacin; RAN, ranitidine; RD-6-L, M, and H represent Ruda-6 at low, medium and high doses, respectively.

To confirm the gastro-protective effects of RD-6, rats undergoing indomethacin were pretreated with RD-6, and stomach tissue was observed. As expected, 6 h of indomethacin challenge caused distinct wound healing in the gastric mucosa compared to the control group, including massive linear and punctate hemorrhagic lesions. In contrast, ranitidine or RD-6 pretreatment successfully alleviated the indomethacin-induced injury, among which RD-6 at 2.7 g/kg had a significant improvement with an inhibition rat of 64.58% (Figure 3A; Table 2). Additionally, the preventive effect of RD-6 was evaluated histologically. H&E staining showed that severe gastric lesions were induced in the model group compared with the control group, such as haemorrhage, epithelial cell loss, submucosal oedema and partial muscle fiber lysis. However, ranitidine or RD-6 markedly relieved these damages, as shown by slight hemorrhage, fewer desquamated epithelial cells, less mucosa and submucosa oedema than the model group (Figure 3B).

**TABLE 2:** Protective effects of RD-6 against GU in rats induced by IND.

Treatments	Dose (g/kg)	Ulcer index	Ulcer area (mm^2^)	Inhibition rate (%)
NC	-	0	0	-
IND	-	78.9 ± 7.4^##^	61.32 ± 11.57^##^	-
IND + RAN	0.04	72.8 ± 6.7	32.96 ± 6.01**	46.25
IND + RD-6-L	0.27	61.5 ± 7.4	28.76 ± 5.82**	53.10
IND + RD-6-M	1.35	61.3 ± 6.9	27.93 ± 7.02**	54.45
IND + RD-6-H	2.7	39.2 ± 9.8**	21.72 ± 5.84**	64.58

Notes: Results were shown as mean ± S.E.M. (*n* = 10). (##) represents the significant differences compared to the NC, group at *p* < 0.01, whereas (**) represents the significant differences compared to the IND, group at *p* < 0.01. NC, normal control; IND, indomethacin; RAN, ranitidine; RD-6-L, M, and H represent Ruda-6 at low, medium and high doses, respectively.

### 3.3 Anti-inflammatory effects of RD-6 in indomethacin-induced GU rats

**FIGURE 4:**
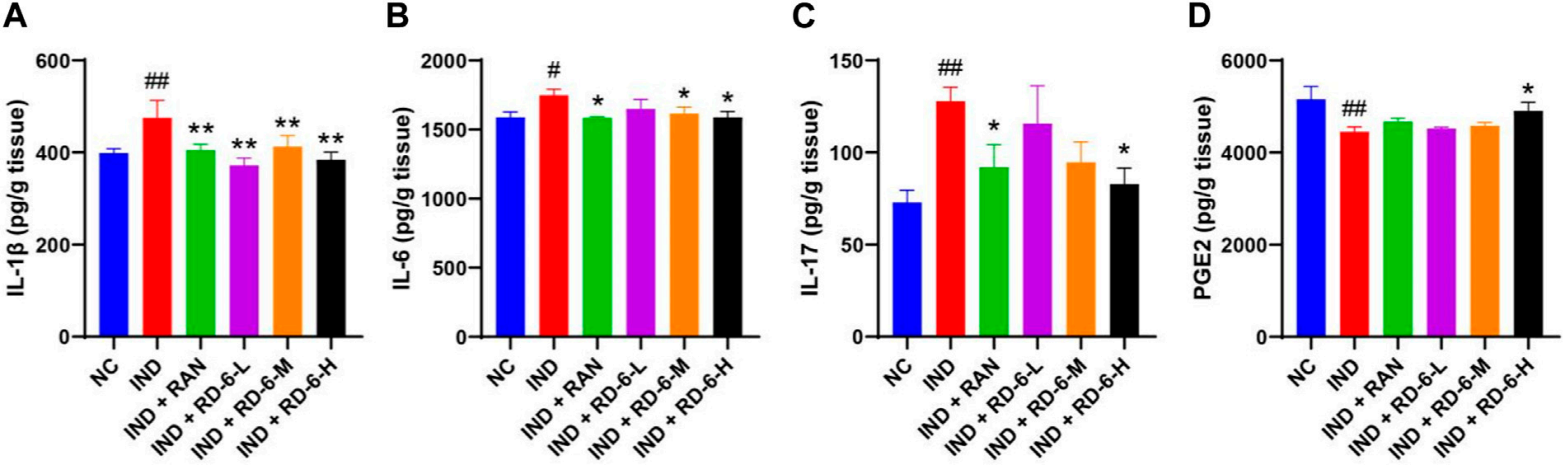
The pretreatment of RD-6 exerts anti-inflammatory effects against indomethacin-induced GU rats. The levels of IL-1β **(A)**, IL-6 **(B)**, IL-17 **(C)**, and PGE2 **(D)** in gastric tissue. Data are expressed as mean ± S.E.M (*n* = 6). One-way ANOVA with the uncorrected Fisher’s LSD test was used to evaluate multiple comparisons. #*p* < 0.05, and ##*p* < 0.01 vs. the NC group; *p < 0.05, and **p < 0.01 vs. the IND group. NC, normal control; IND, indomethacin; RAN, ranitidine; RD-6-L, M, and H represent Ruda-6 at low, medium and high doses, respectively.

According to the predicted targets and pathways of network analysis, the levels of inflammation-associated parameters including IL-1β, IL-6, IL-17 and PGE2 in gastric homogenate were examined. The ELISA analysis showed that indomethacin significantly increased the contents of IL-1β, IL-6, and IL-17 (*p* < 0.05), while pretreatment with ranitidine or RD-6 essentially inhibited the production of these inflammatory cytokines (*p* < 0.05) (Figures 4A–C). Moreover, the level of PGE2 in gastric tissue was obviously decreased during GU progression compared with the control group (*p* < 0.01). However, the high dose of RD-6 enhanced the PGE2 content (*p* < 0.05) (Figure 4D).

### 3.4 RNA-seq analysis identified differential expressed genes and signaling pathway in RD-6 treated rats

**FIGURE 5:**
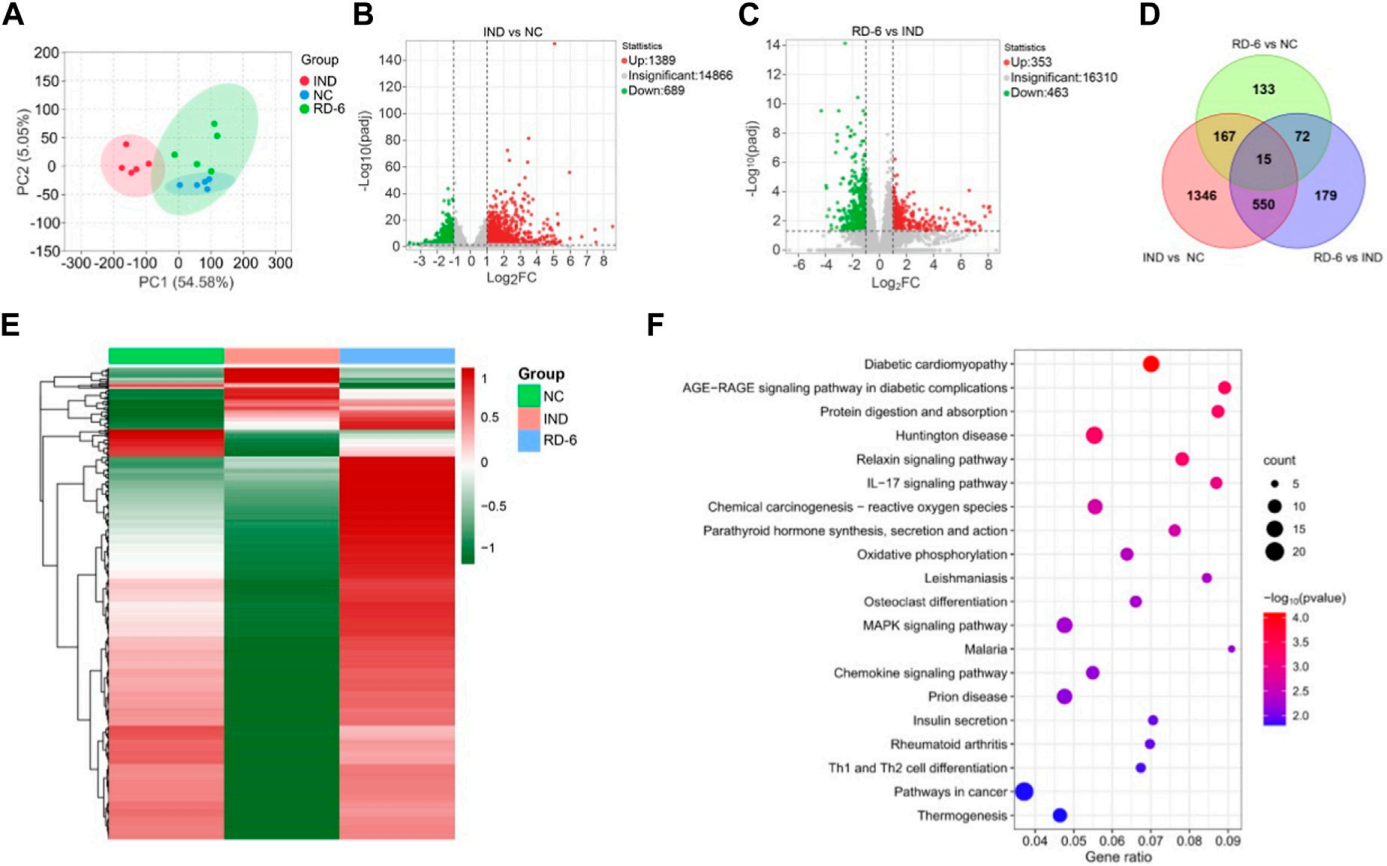
RNA-Seq analysis of the gastric tissue of RD-6-pretreated IND-induced GU rats. **(A)** PCA analysis among three groups. **(B)** Volcano plots of DEGs between IND vs. NC groups. **(C)** Volcano plots of DEGs between the IND group and the IND plus RD-6-pretreated group. **(D)** Venn diagram of DEGs among three groups. **(E)** Hierarchical clustering heatmap of DEGs. The redder the color, the higher the expression; the greener the coloer, the lower the expression. **(F)** KEGG enrichment analysis for DEGs. The size of the circles represents the number of genes and the color represents the *p*-value. NC, normal control; IND, indomethacin; RD-6, Ruda-6.

To more thoroughly understand the molecular mechanism of RD-6 on GU prevention, differential genes and pathways in rat gastric tissue were explored through RNA-Seq technology in the NC, IND and RD-6-H groups. The correlation heatmap of gene expression levels among groups displayed that the experimental design was reliable and the sample chosen was reasonable (Supplementary Figure S2). Besides, PCA analysis showed that the RD-6 group was significantly separated from the model group, and close to the control group (Figure 5A). By applying the cutoffs of padj <0.05 and |Log2FC| ≥ 1, a total of 2078 DEGs were identified in IND vs. NC groups, 1,389 of which were upregulated and 689 were downregulated (Figure 5B); 816 DEGs were found between the IND group and the IND plus RD-6-pretreated group, including 353 up- and 463 downregulated genes (Figure 5C). A Venn diagram of the number of DEGs is shown in Figure 5D, and it was found that 565 out of 816 DEGs induced by indomethacin were also associated with the RD-6 pretreatment. The expression patterns of these 565 DEGs in three experimental groups are listed in Supplementary Table S7. In the advanced analysis, a hierarchical clustering heatmap of DEGs vividly showed that RD-6 significantly restored the expression level of most of the dysregulated genes, which were the target genes to be sought (Figure 5E). Furthermore, to investigate the potential pathways involved in anti-GU, the KEGG enrichment analysis of these DEGs was performed. In total, 36 KEGG pathways were significantly enriched (*p* < 0.05) (Supplementary Table S8). Among them, the top 20 most significant signaling pathways were selected to visualize, including the IL-17 signaling pathway, the MAPK signaling pathway and pathways in cancer (Figure 5F). Interestingly, the IL-17 pathway was also significantly enriched in the network analysis.

### 3.5 RD-6 regulated the IL-17 signaling pathway and potential targets

**FIGURE 6:**
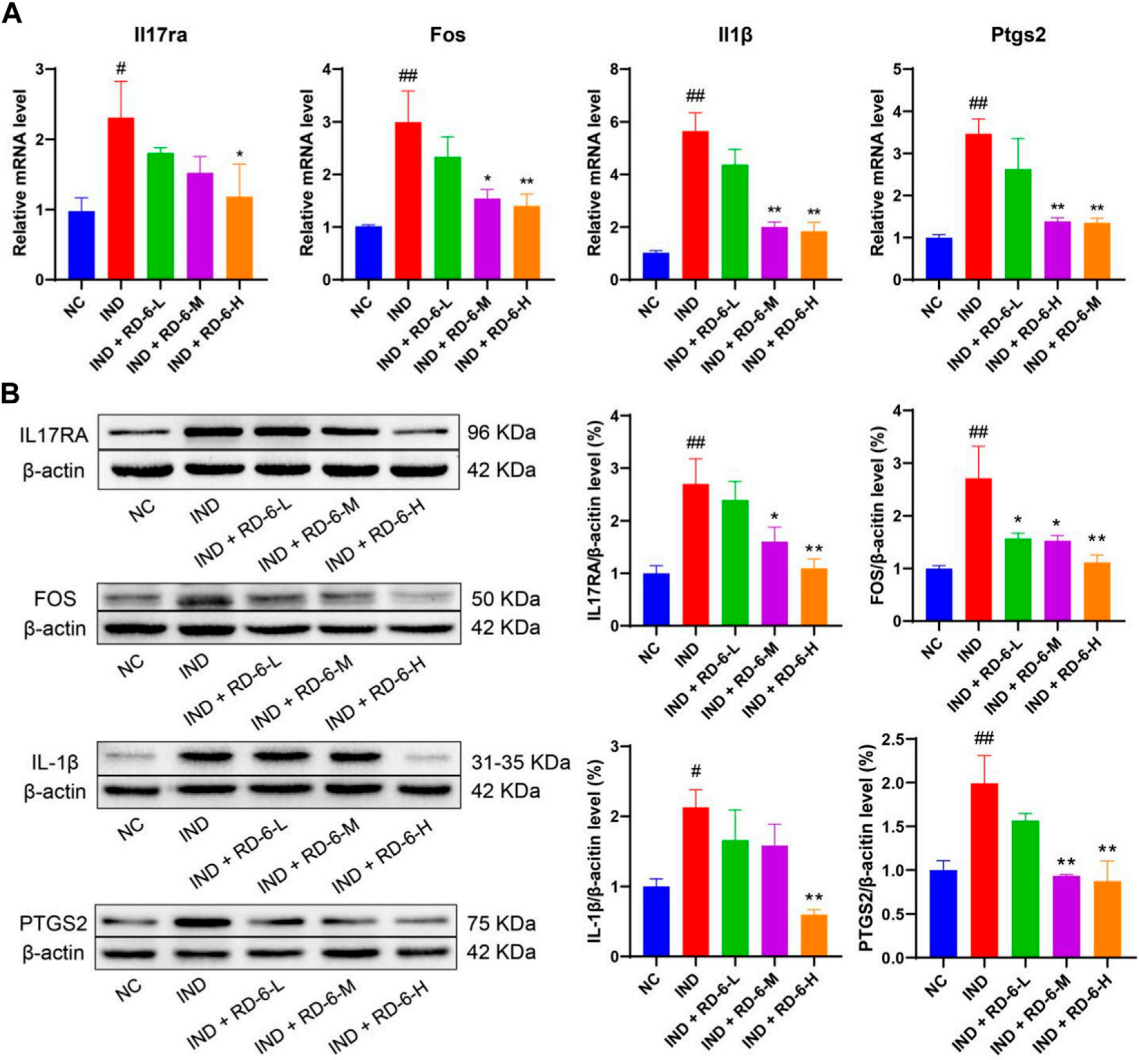
The pretreatment of RD-6 inhibited the IL-17 signaling pathway in indomethacin-induced GU rats. The expression of IL17RA, FOS, IL1B, and PTGS2 determined in gastric tissue by qRT-PCR **(A)** and western bloting **(B)**. Data are expressed as mean ± S.E.M (*n* = 3). One-way ANOVA with the uncorrected Fisher’s LSD test was used to evaluate multiple comparisons. #*p* < 0.05, ##*p* < 0.01 vs. NC group; *p < 0.05, **p < 0.01 vs. IND group. NC, normal control; IND, indomethacin; RD-6-L, M, and H represent Ruda-6 at low, medium and high doses, respectively.

Next, four potential targets of the IL-17 signaling pathway, IL17RA, FOS, IL1B and PTGS2, were selected to verify the results further. As shown in Figure 6A, the mRNA expression of Il17ra, Fos, Il1b, and Ptgs2 was significantly upregulated in the IND group compared with rats in the NC group (*p* < 0.05). While different doses of RD-6 administration evidently converted these changes, with a reduction of Il17ra, Fos, Il1b and Ptgs2 levels (*p* < 0.05). In line with the mRNA expression results, indomethacin dramatically increased the protein levels of IL17RA, FOS, IL1B and PTGS2 compared with the NC group (*p* < 0.05). However, when animals were pretreated with RD-6, the levels of IL17RA, FOS, IL1B and PTGS2 were visibly inhibited compared to those in the ulcerated rats (Figure 6B) (*p* < 0.05).

## 4 Discussion

GU is a common digestive system disease characterized by inflammatory cell infiltration and inflammatory cytokines secretion (Zhou et al., 2023). Traditional Mongolian medicine formulae RD-6 has many years of clinical application experience in treating digestive system diseases such as GU. Herein, for the first time, we evaluated the anti-inflammatory properties of RD-6 in the process of alleviating GU and its effect on the IL-17 signaling pathway from the perspective of network analysis and RNA-Seq.

The efficacy of natural medicine often depends on its numerous chemical components. The network analysis method provides convenience for predicting and screening their effectiveness. According to our previous studies on the components of RD-6 (Feng et al., 2023), potential anti-GU active components of RD-6 were found using the TCMSP database, including flavonoids (quercetin, luteolin, and naringin), alkaloids (piperine), and terpenes (dehydrocostus lactone). The anti-ulcer or anti-inflammatory effect of these ingredients has been confirmed. For example, quercetin can inhibit the inflammatory reaction caused by indomethacin, thus protecting the gastrointestinal tract (Carrasco-Pozo et al., 2016; Boonyong et al., 2023). Luteolin and its glycoside derivatives play an important role in inflammatory diseases by reducing the expression of pro-inflammatory factors (IL-6 and IL-1β) (Attiq et al., 2021) and increasing the production of the anti-inflammatory factor (IL-10) (Okeke and Uzonna, 2019). There is evidence that naringin inhibited the levels of inflammatory factors, TNF-α, IL-6 and IL-8, and effectively reduced the ethanol-induced inflammatory reaction, thus protecting mice from gastric injury (Li et al., 2021). Piperine and dehydrocostus lactone can effectively prevent ethanol-induced gastric mucosal injury (Zheng et al., 2016; Duan et al., 2022). Overall, six hypothetically bioactive compounds, namely, quercetin, luteolin, naringenin, formononetin, piperine and dehydrocostus lactone were identified by network analysis, but there is no pharmacological evidence for this based on the data in this study.

Indomethacin has a higher ulcerogenic ability than other NSAIDs; thus, it has been considered as the inducer choice for GU models (Suleyman et al., 2010). In this study, oral administration of indomethacin (30 mg/kg) caused an increase in inflammatory factors and ulcer index in rats compared with the control group. Our results are consistent with previous research results, that is, indomethacin can cause gastric mucosal injury and even GU by causing an inflammatory reaction (Ogaly et al., 2021; Bao et al., 2022). However, the ulcer manifestations were alleviated when the animals were pretreated with ranitidine or RD-6. Especially the RD-6-H (2.7 g/kg) group was superior to other pretreatment groups with a lower ulcer index and a higher ulcer inhibition rate. In addition, the histopathological examination of gastric tissue in the GU model group showed that indomethacin caused obvious ulcer injury and inflammatory cell infiltration. These observed changes were consistent with the significant accumulation of inflammatory indicators (IL-1β, IL-6 and IL-17) in gastric tissue. Nevertheless, the relief of ulcer injury and the inhibition of the overexpression of the above inflammatory factors by RD-6 suggested that the anti-inflammatory activity of RD-6 is involved in the gastric protection of RD-6.

A joint analysis of the network prediction approach and RNA-Seq revealed that the inhibitory mechanism of RD-6 for GU may involve multiple pathways, such as the AGE-RAGE signaling pathway, pathways in cancer, and the IL-17 signaling pathway. Studies have shown that these signaling pathways are closely related to the GU process. For example, the interaction of AGE-RAGE can cause neuronal damage, result in the subsequent activation of the inflammatory signal cascade and intensify oxidative stress, further leading to various neurological disorders and inflammatory-related diseases (Dong et al., 2022; Reddy et al., 2023). Salama et al. (2023) found that the protective effects of dapagliflozin (a drug clinically used to treat type 2 diabetes) on ethanol-induced gastric injury in rats were associated with a significant reduction in RAGE levels. Moreover, excessive expression of inflammatory factors is associated with tumor promotion, which may increase the risk of gastric cancer (Fei et al., 2004). It has been recently shown that IL-17 is associated with mucosal atrophy and gastric precancerous lesions (Li et al., 2021; Qian et al., 2022). In the present study, indomethacin treatment increased the IL-17 level, indicating the risk of GU developing into gastric cancer. RD-6 may alleviate the symptoms of gastric inflammation and ulcers and even prevent their progression to gastric cancer by regulating these signaling pathways.

It is worth mentioning that the IL-17 signaling pathway is very important in the anti-GU process according to our network pharmacological prediction results, and the RNA-Seq results also suggested that the DEGs between the NC, IND and RD-6 groups were significantly enriched in the IL-17 signaling pathway. As a cytokine secreted by immune cells, IL-17 has the ability to extensively induce inflammatory reactions. IL-17 can recruit neutrophils, thus leading to inflammation by promoting the production of multiple cytokines. A study by Shiomi et al. (2008) reported that IL-17 was involved in gastritis and neutrophil infiltration caused by *H. pylori* infection. Our animal experiment results were similar to that. Indomethacin caused inflammatory cell infiltration, while IL-17 content was upregulated in rat gastric tissue. Therefore, we speculate that IL-17 and its signaling pathway are a potential mechanism for RD-6 against GU. To verify this hypothesis, the expression changes of key molecules of the IL-17 signaling pathway were detected at the mRNA and protein levels. Our results showed that the mRNA and protein levels of IL-17RA, FOS, IL-1B and PTGS2 in the gastric tissue of the GU group were upregulated when compared with the control group. IL-17RA is a key molecule in the IL-17 signaling pathway and is also one of the DGEs in our RNA-Seq analysis. IL-17RA is one of the members of the IL-17 receptor family (IL17RA-IL17RE), and plays a vital role in cellular processes, including inflammation and cancer cells (Goepfert et al., 2022). A clinical study found that IL-17RA rather than the other four members of the IL-17 receptor family is significantly upregulated in human gastric cancer tissues compared with normal gastric tissues (Jiang et al., 2015). As the downstream molecule of the IL-17 signaling pathway, transcription factor FOS is a member of the activator protein 1 (AP-1) family (Li et al., 2016). FOS participates in cell cycle events, including proliferation and differentiation (Liu et al., 2023; Oh et al., 2023). Studies have found that the expression level of the FOS gene was increased in GU model animals induced by ethanol and indomethacin (Ganguly and Swarnakar, 2012; Basatinya et al., 2021). FOS is then transferred into the cellular nucleus and increases the transcriptional activation of some inflammatory genes, such as IL-1B, PTGS2. Based on the above analyses, indomethacin activated the downstream signal transduction of IL-17 in gastric tissue. However, the mRNA and protein levels of IL-17RA, FOS, IL-1B and PTGS2 in the RD-6 pretreatment groups were downregulated compared with the model group. All these evidences strongly suggest that RD-6 has a potential anti-inflammatory effect by inhibiting the IL-17 signaling pathway in the anti-GU process.

## 5 Conclusion

In sum, by combining network analysis, RNA-Seq and *in vivo* validation, we confirmed that RD-6 can ameliorate the gastric ulcer induced by indomethacin. Meanwhile, RD-6 can mediate the IL-17 signaling pathway by inhibiting IL17RA, FOS, IL1B and PTGS2 to relieve inflammatory factors in GU rats. Accordingly, our study proved the potential gastroprotective role of RD-6 in GU-related inflammation.

## Data Availability

The datasets presented in this study can be found in online repositories. The names of the repository/repositories and accession number(s) can be found below: NCBI BioProject (https://www.ncbi.nlm.nih.gov/bioproject/), PRJNA944205.
