# Supplementary material for: An integrated network analysis, RNA-seq and in vivo validation approaches to explore the protective mechanism of Mongolian medicine formulae Ruda-6 against indomethacin-induced gastric ulcer in rats
Source: Front Pharmacol. 2023 Aug 10;14:1181133. doi: 10.3389/fphar.2023.1181133 (PMC10449537; doi:10.3389/fphar.2023.1181133)
Supplement: Supplementary file 1 [file DataSheet1.docx]

Supplementary Material

An integrated network analysis, RNA-Seq and in vivo validation approaches to explore the protective mechanism of Mongolian medicine formulae Ruda-6 against indomethacin-induced gastric ulcer in rats

Lan Feng, Lisha A, Terigele Bao, Xiyele Mu, Na Ta, Qiang Duan, La Ta, Yongsheng Chen, Laxinamujila Bai * and Minghai Fu *

*** Correspondence:** Laxinamujila Bai, namujila@126.com; Minghai Fu, m.fu@hainmc.edu.cn

# Supplementary Figures


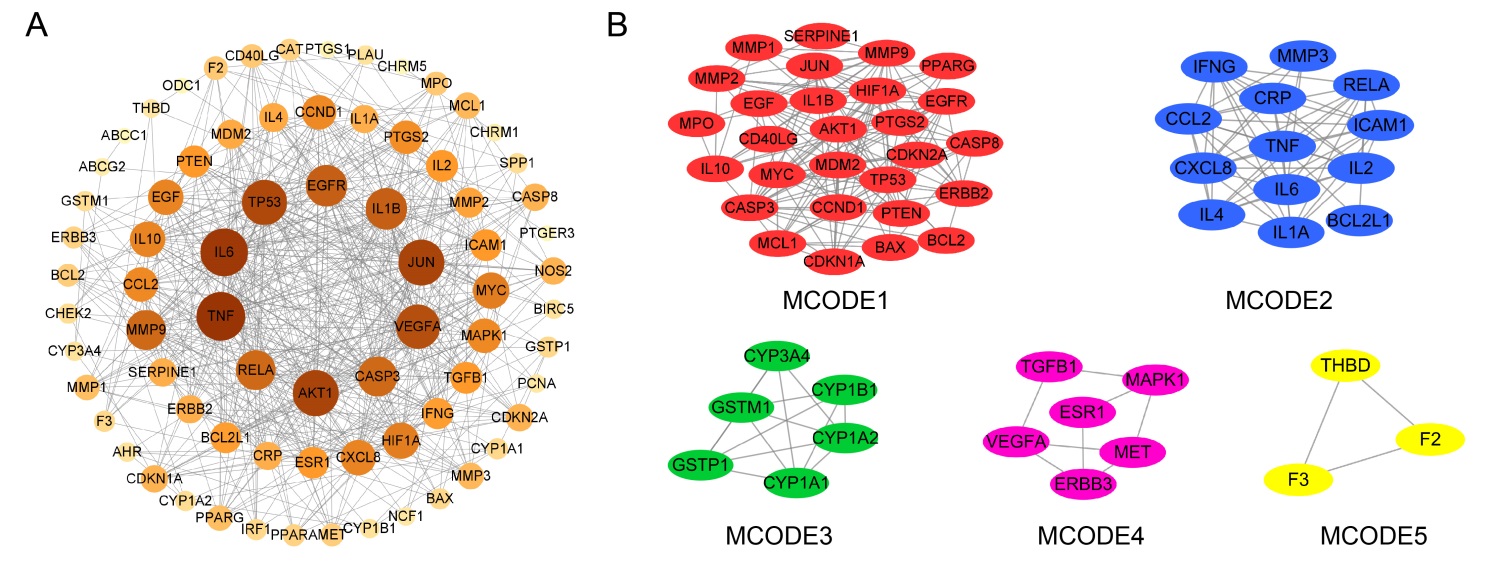


**Supplementary Figure 1.** The PPI analysis of the common targets between RD-6 and GU. (A) PPI network. The color is proportional to its degree value. The redder the node, the more important the target in the network. (B) The cluster analysis of PPI network based on MCODE. The 75 overlapped target gene were divided into 5 clusters. MCODE1 (red): 28 nodes; MCODE2 (blue): 13 nodes; MCODE3 (green): 6 nodes; MCODE4 (purple): 6 nodes; MCODE5 (yellow): 3 nodes.

**
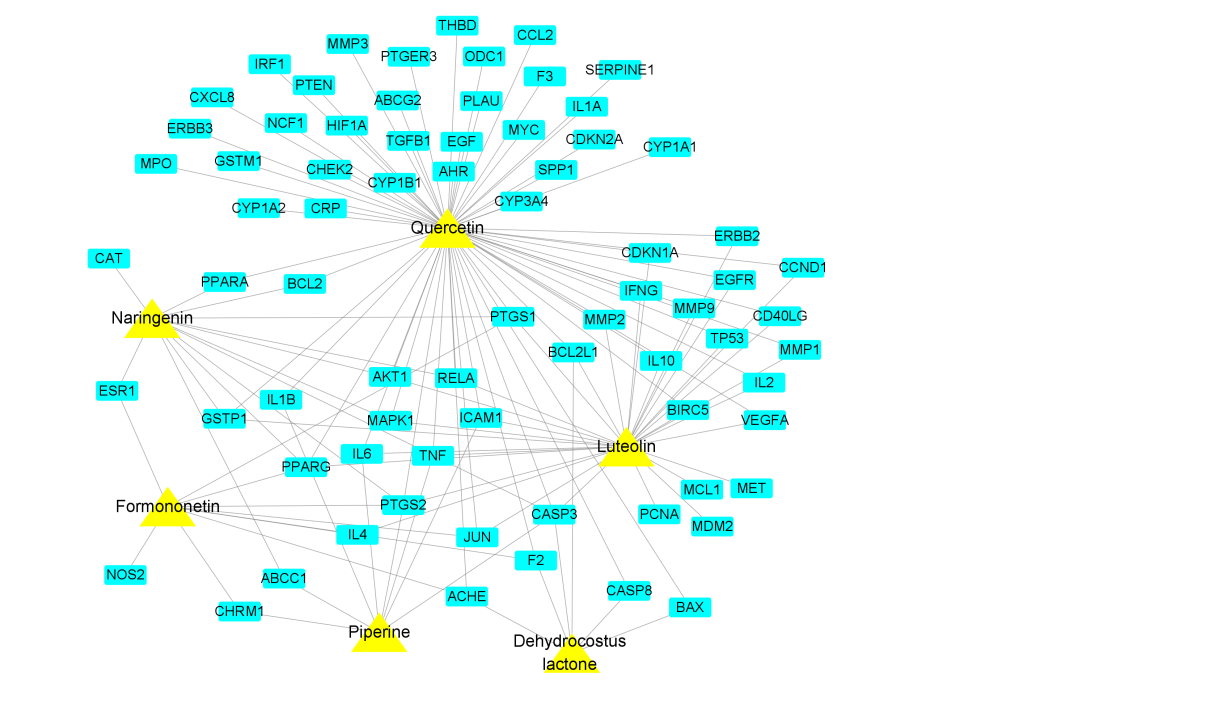
**

**Supplementary Figure 2.** Compound-target network by Cytoscape 3.8.0. The red diamond represents gastric ulcer; the yellow circle represents the potential active components of RD-6; the purple hexagon represents the Mongolian medicine formulae RD-6; the green rectangle represents the potential targets.


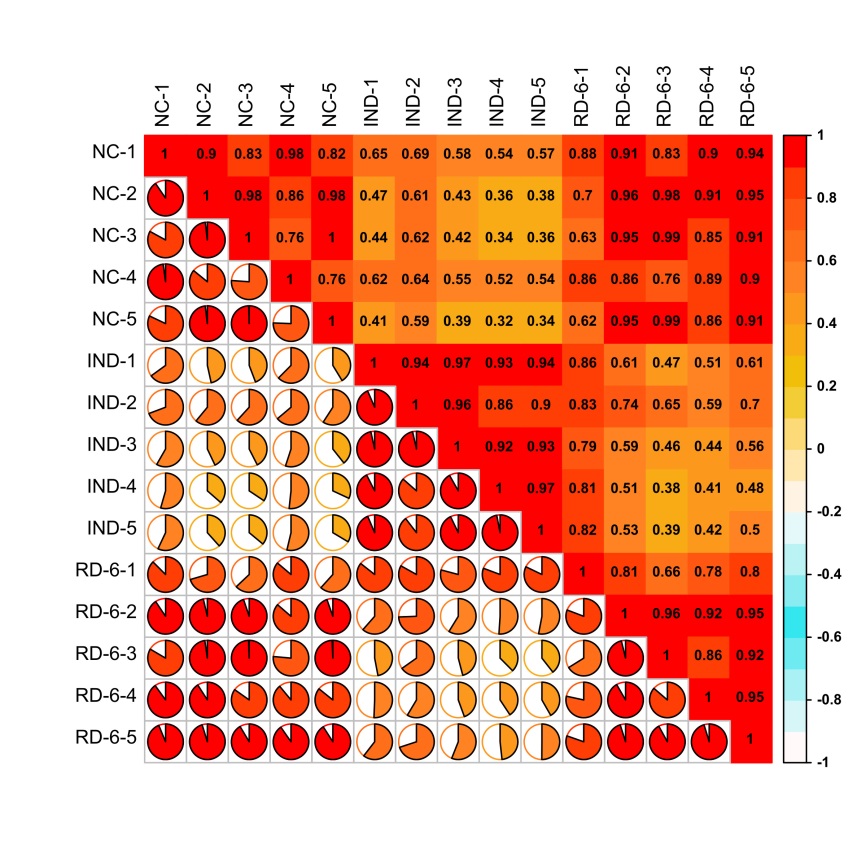


**Supplementary Figure 3.** Correlation coefficient diagram and heatmap between samples based on RNA-Seq. Positive correlation is in red and negative correlation is in blue.
